# Evaluation of the expression of the oncogen C-ERBB-2/HER2 in advanced gastric cancer cases from Costa Rica

**DOI:** 10.3332/ecancer.2019.962

**Published:** 2019-09-19

**Authors:** Eugenia Cordero-García, Andrés Baéz-Astúa, Yolanda Roa-Martínez, Vanessa Ramírez-Mayorga, Warner Alpízar-Alpízar

**Affiliations:** 1 Toxicology and Drug Dependence, Department of Pharmacology, School of Pharmacy, University of Costa Rica, 11501-2060 San José, Costa Rica; 2 Institute of Pharmaceutical Research (INIFAR), University of Costa Rica, 11501-2060 San José, Costa Rica; 3 Molecular Oncology Laboratory, Calderón Guardia Hospital, Caja Costarricense del Seguro Social (CCSS), San José, 10101 Carmen, Aranjuez, Costa Rica; 4 Pathology Service, Calderón Guardia Hospital, Caja Costarricense del Seguro Social (CCSS), San José, 10101 Carmen, Aranjuez, Costa Rica; 5 Cancer Epidemiology Program, Institute for Health Research (INISA), University of Costa Rica, 11501-2060 San José, Costa Rica; 6 Public Nutrition Section, School of Nutrition, University of Costa Rica, 11501-2060 San José, Costa Rica; 7 Center for Research in Microscopic Structures (CIEMIC), University of Costa Rica, 11501-2060 San José, Costa Rica; 8 Department of Biochemistry, School of Medicine, University of Costa Rica, 11501-2060 San José, Costa Rica

**Keywords:** oncogene, gastric cancer (GC), immunohistochemistry (IHC), fluorescent in situ hybridisation (FISH), HER2

## Abstract

**Justification:**

The prevalence of gastric cancer (GC) with increased expression of the HER2 oncoprotein shows important variations worldwide. Incidence and mortality rates of GC in Costa Rica are among the highest in Latin America and the world; however, the prevalence of HER2-positive cases in this country is unknown. Evaluation of this parameter is important to decide the therapeutic approach for GC patients. The aim of this study was to provide an estimation of the prevalence of GC patients overexpressing the HER2 oncogene in Costa Rica.

**Methods:**

The investigation was carried out in two phases. The first one consisted of a retrospective review of 331 clinical records of patients diagnosed with advanced or metastatic GC from January 2010 to January 2012 in four hospitals in Costa Rica. In the second phase, immunohistochemistry (IHC) and fluorescent *in situ* hybridisation (FISH) analyses were performed in formalin-fixed and paraffin-embedded (FFPE) surgical samples from 50 patients diagnosed with GC between 2012 and 2015.

**Results:**

Of the 331 clinical files reviewed, the assessment of HER2 status was carried out in 62 patients (18.7%), of which only five (8%) were HER2-positive. In the 50 surgical specimens in which IHC and FISH analyses were performed, two of them (4%) presented overexpression and amplification of the HER2 oncogene.

**Conclusion:**

This study suggests that the prevalence of GC cases overexpressing the HER2 oncogene in Costa Rica is less than 8%. This is the first attempt ever undertaken to estimate the prevalence of HER2-positivity in GC in Costa Rica.

## Introduction

Gastric cancer (GC) is the fifth most diagnosed neoplasm and the third cause of cancer death worldwide. In 2018 alone, more than 1 million new cases and 782,000 deaths were attributed to this type of malignant tumour [1–3]. In Costa Rica, GC is the fifth highest incident tumour and the second cause of cancer death in both sexes [3–6]. Although GC incidence in this country has decreased over recent years, its mortality is still high, mainly because most of the cases are diagnosed at advanced stages. Consequently, the disease is often hard to control. Costa Rica does not have an early screening GC programme that covers the entire population. There is only one such programme located in Cartago, the province with the highest number of GC cases in the country [7]. Although this initiative has proven effective [7], extending the programme nationwide is not feasible in the near future due to cost.

In 2010, the first prospective, randomised, multicentre phase III study was published (the ToGA study), in which the efficacy and safety of the monoclonal antibody, Trastuzumab was evaluated in advanced gastric adenocarcinoma and cancer of the gastroesophageal junction overexpressing human epidermoid growth factor receptor 2 (HER2) [8]. This study demonstrated that administration of the antibody, in combination with chemotherapy, results in a significant increase in overall and progression-free survival of patients, compared to patients only receiving chemotherapy [8]. It was based on this study that the Food and Drug Administration (FDA) and the European Medicines Agency (EMA) approved the use of Trastuzumab in patients with advanced GC positive for HER2 [8–12].

Functional studies performed in cancer with HER2 overexpression have shown that HER2 binds to other receptors of the epidermal growth factor receptor (EGFR) family, thus resulting in dimerisation and phosphorylation of these, even in the absence of ligands [13–14]. Consequently, dimerisation of these tyrosine kinase receptors leads to the activation of intracellular signalling cascades, mainly involving the PI3K/Akt, Ras/Raf/MAPK and PLC/PKC pathways [13–16]. Given the diversity of cellular responses elicited as a result of the activation of these pathways, HER2 has been associated with the activation of a series of processes driving cancer progression, such as proliferation, migration, invasiveness, angiogenesis, resistance to apoptosis and metastatic potential [13–16].

The prevalence of GC cases with increased expression of the HER2 oncoprotein presents substantial variations worldwide, ranging between 2% and 45% [17]. This wide variation could be attributed to several aspects, including histological subtype and anatomical location of the tumour, the subjectivity of the criteria employed for the interpretation of the tests, the heterogeneity in the expression of the receptor and the ethnic background of the population. In general, HER2 is overexpressed mainly in malignant lesions located in the gastroesophageal junction and stomach cardias. Also, the expression of this oncogenic protein tends to be higher in malignant tumours of the intestinal histological subtype and advanced stages of the disease [9–11, 13, 17].

The methods accepted by FDA and EMA for the determination of HER2 status are immunohistochemistry (IHC) analysis and *in situ* hybridisation with fluorescence-labelled probes (FISH) [10]. The scoring system used for HER2 assessment in GC was developed by Hoffman *et al* [18]. According to this system, HER2 immunoreactivity in neoplastic tissue samples varies from 0 to 3+. More specifically, the cases in which IHC staining is negative or barely perceptible (0 to 1+) are considered negative for HER2; those with strong staining (IHC 3+) in more than 10% of cancer cells are regarded as positive. The IHC is considered inconclusive when HER2 immunoreactivity in basolateral or lateral membranes of more than 10% of cancer cells is weak to moderate (IHC 2+). It is in these particular cases when the FISH analysis is used as a confirmatory test to reach a final conclusion as to whether the patient is regarded as HER2-positive or negative [18].

In Costa Rica, no studies have been conducted to determine the prevalence of GC cases with increased expression of HER2. The determination of the prevalence of malignant lesions with overexpression of HER2 in Costa Rican GC patients is important to have a clearer understanding of the relevance of this genetic alteration in the aetiology of the disease, as a potential prognostic biomarker and as a criterion to decide the therapeutic approach of the patients. Since the prevalence of HER-positivity in Costa Rica is not known, the anti-HER2 agents have not been employed so far for the treatment of GC, which means that HER2-positive GC patients are not deriving clinical benefit from these pharmacological agents. In this study, we provide for the first time an estimation of the prevalence of Costa Rican GC patients overexpressing the HER2 oncogene.

## Methods

The investigation was carried out in two phases. The first part consisted of a retrospective review of clinical records of patients diagnosed with advanced or metastatic GC in public Costa Rican hospitals between 2010 and 2012. Through this revision, we identified the patients who underwent evaluation of HER2 status. In the second phase, IHC and FISH tests were performed on formalin-fixed and paraffin-embedded (FFPE) surgical tissue samples from 50 patients diagnosed with GC at the Calderón Guardia Hospital between 2012 and 2015. The study was approved by the Scientific Ethics Committee of the University of Costa Rica (# 817-B2-371), Institutional Scientific Ethics Committee (R013-SABI-00048) of the Caja Costarricense del Seguro Social (CCSS) and Local Bioethics Committee of the Calderón Guardia Hospital (CLOBI-01 January 2015).

## Retrieval of information from clinical records

We retrospectively reviewed the clinical records of 331 patients older than 18 years that were diagnosed with resectable advanced or metastatic GC between January 2010 and January 2012 in the four public hospitals having Clinical Oncology service at the time of the study; namely, Max Peralta Hospital, Mexico Hospital, Calderón Guardia Hospital and San Juan de Dios Hospital. From each file, we retrieved the following information: anatomical location of the tumour, histological subtype and HER2 status (positive, negative, not determined). In those cases, in which the evaluation of HER2 had been made, the date of completion and the type of test carried out (IHC and/or FISH) were documented. The information collected was processed, digitised and managed in databases in Excel files (Microsoft Office).

### Calculation of the sample

The sample required to carry out this study was determined according to the historical data (3 years: 2008–2010) of the number of GC cases diagnosed per year in Costa Rica, and the number of GC cases treated per year. The statistical power for the present study is 80% (α = 0.05).

### Standardisation and validation of the IHC and FISH tests for HER2

In addition to generating an estimation of the prevalence of positive cases for HER2, we standardised and validated the tests for the determination of the HER2 status in GC [9]. To do this, 50 FFPE surgical samples of GC were retrieved from the tissue archive of the Department of Pathology, Calderón Guardia Hospital. This material was not randomly selected; most of the procured tissue samples were from advanced GC tumours, located in the proximal region of the stomach and of intestinal histological subtype. These characteristics, according to the literature, increase the probability of finding overexpression of the HER2 oncoprotein [9–11, 13, 17].

### Immunohistochemistry staining and scoring

The 5-μm-thick FFPE tissue sections were deparaffinised with xylene and hydrated in gradual series of ethanol–water dilutions. HER2 immunostaining was carried out with the HercepTestTM kit (Dako, Denmark, code: K520421) following the manufacturer’s instructions, with minor adjustments in the antigen retrieval and incubation time of the anti-HER2 primary antibody. Heat-induced epitope retrieval in a T/T Micromed microwave processor (Milestone, Sorisole, Italy) was performed at 98 °C for 15 min in the antigen retrieval solution provided by the kit. The primary antibody was applied to the tissue sections and incubated overnight at 4 °C. Evaluation of the stainings was made in an Olympus BX43 microscope by two of the researchers participating in the study (WAA and ECG). A third observer (YRM), an experienced pathologist who also collaborated in this study, independently evaluated the sections.

### Fluorescence in situ hybridisation and evaluation

FISH was performed by using the HER2 FISH pharmDxTM kit (Dako, code K5331/K5599), kindly donated by the company MAKOL (Costa Rica), following the manufacturer’s instructions with some modifications. Specifically, the tissue sections were placed on a heat plate for 30 min at 37 °C to facilitate the deparaffinisation process. Subsequently, the slides were immersed in xylene and hydrated in a gradual series of ethanol–water dilutions. The FISH tests were evaluated on a Nikon Eclipse 80i microscope (Molecular Oncology Laboratory, Calderón Guardia Hospital) between 30 min and 24 h after finalising the experimental procedure. This evaluation was carried out by two of the researchers participating in the study (ABA and ECG). GC cases showing amplification of the gene encoding HER2 (HER2/CEN17 ratio > 2) were considered HER2-positive; those in which the ratio was less than 2 were regarded as negative.

### Positive and negative controls for IHC and FISH tests

As experimental controls, we selected three FFPE tissue blocks from breast cancer in which IHC and FISH for HER2 had been previously performed. Specifically, one of the tissue samples was HER2-positive (score 3+) and two were negative (score 0). Tissue sections of these blocks were included each time we performed an IHC staining for GC samples. Similarly, control slides provided by the manufacturers as part of materials of the HER2 FISH pharmDxTM kit were included each time we performed FISH experimental procedures.

## Results

### Prevalence of HER2-positive gastric cancer cases in clinical files

For the present investigation, a total of 331 clinical records were reviewed. According to these files, only 62 of the patients were subjected to HER2 IHC/FISH analysis, and five of them (8%) were HER2-positive. Of these five patients, two had a malignant lesion located in the stomach body, one in the antrum and two in the body-antrum. According to histological criteria, three tumours were classified as well-differentiated and two as moderately differentiated; that is, five cases were of the intestinal subtype according to Lauren’s scheme. Regarding the TNM stage at the time of diagnosis, two patients corresponded to stage II-A and three to stage II-B. The average age of the five patients was 73 years (range 66–80 years) (Table 1).

### Immunohistochemistry and fluorescent in situ hybridisation analyses for HER2

**Figure 1.**
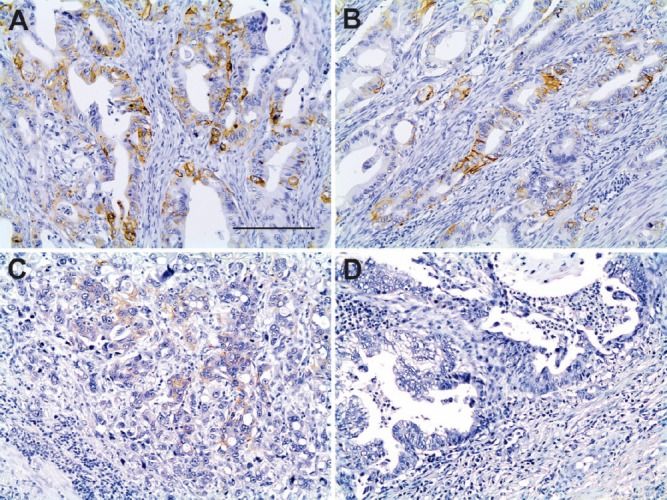
Immunohistochemistry for HER2 in tissue samples of advanced GC. (A) and (B): Positive IHC 3+ staining (brown) of the basolateral or lateral cell membrane. (C): Barely visible IHC 1+ staining. (D): Absence of IHC membrane staining. Scale bar: A–D ≈ 100 μm.

Table 2 summarises some clinicopathological characteristics of 50 patients from whom the neoplastic tissue was retrieved and used to perform IHC and FISH tests. Strong HER2 staining in cancer cells was observed in 2 (4%) out of the 50 samples analysed by IHC. The HER2-expressing cancer cells formed clusters that were heterogeneously distributed in the malignant lesion, adjacent to HER2-negative areas. HER2 staining was mostly seen in the basolateral and/or lateral membrane of the neoplastic cells (Figure 1A and B). According to the scoring criteria used to quantify the expression of HER2 by IHC in GC, strongly positive staining in the membrane of at least 10% of cancer cells is assigned the highest score (3+) [18]. In accordance with these criteria, the two cases described above corresponded to patients with unequivocal overexpression (3+) of HER2 (Figure 1A and B). In two other cases, barely perceptible immunoreactivity was observed in the patches of tumour cells that corresponded to less than 10% of the total malignant cell population, which according to the classification criteria corresponded to HER2 1+ (Figure 1C). The remaining 46 samples were negative for HER2 (Figure 1D).

**Figure 2.**
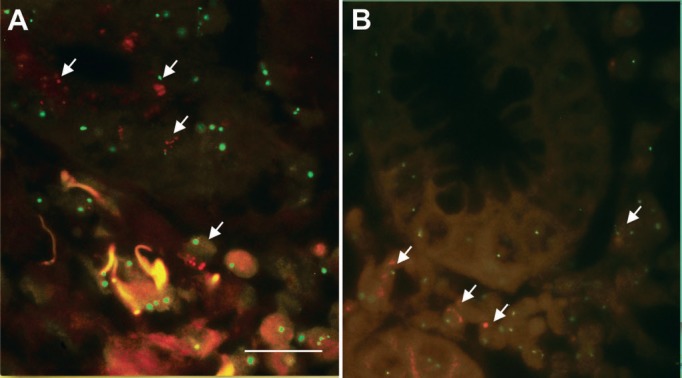
Fluorescent in situ hybridisation in tumour cells of advanced GC. (A): The green signal corresponds to chromosome 17 (CEN 17) and the red signal to the gene encoding HER2 amplification of the ERBB2 gene (HER2) (40X). (B): Normal HER2/CEN 17 ratio. Scale bar: A and B ≈ 50 μm.

When performing FISH tests in the 50 tissue samples of GC lesions, 100% concordance between them and the IHC was observed. Specifically, amplification of the gene encoding HER2 was observed in the two samples showing overexpression of the HER2 oncoprotein in the IHC analysis (Figure 2A). The values of the HER2/CEN 17 ratio were greater than 2 in both samples, which according to the standard criteria is considered as amplification of the HER2 oncogene. In the remaining 48 cases, the FISH analysis did not reveal the amplification of the gene coding for HER2 (Figure 2B).

Finally, IHC and the FISH were repeated in the two GC samples showing overexpression and amplification for HER2, respectively. This was done independently by a second investigator (ABA) in the facilities of the Molecular Oncology Laboratory at the Calderón Guardia Hospital, obtaining the same result as in the initial determinations, thus confirming the veracity and reproducibility of the IHC and FISH analyses.

## Discussion

Overexpression of HER2 in malignant gastric tumours has been described in multiple studies, [8, 10, 18, 19]; however, the prevalence of HER2-positive GC cases varies between geographical regions. In Costa Rica, GC is a highly incident malignant tumour and an important cause of cancer death. Also, the prevalence of GC cases overexpressing HER2 in this Latin American country was unknown. This study, therefore, represents the first attempt to characterise the expression pattern and provide an estimation of the prevalence of HER2 in advanced GC in Costa Rica.

According to our review of 331 clinical records, only 62 (18.7%) of the patients diagnosed with advanced GC underwent IHC and FISH analysis, and 8% of those overexpressed HER2. Importantly, according to the accessed clinical records, more than 81% of the 331 patients were not subjected to IHC or FISH analysis, which is due to the fact that these analyses are not routinely carried out for GC in Costa Rica. Although our study provides an estimation that is in agreement with the prevalence reported in other countries [20, 21], it may not be fully representative because of the low number of GC cases in which IHC and FISH test were performed. Also, it is important to point out that the retrieved clinical files represent a specific period (2010 and 2012). Therefore, caution must be exercised when interpreting the present results.

In an attempt to better define the prevalence of advanced GC cases overexpressing HER2, IHC and FISH analyses were carried out in postoperative tissue samples from 50 patients. The IHC revealed overexpression in 2 of the 50 cases, which corresponds to 4%. These two HER2-positive cases corresponded to the intestinal histological subtype of GC. According to the literature, the HER2 oncoprotein tends to be more frequently overexpressed in intestinal subtype [10, 12]. Also, a higher prevalence of HER2-overexpressing cases is generally found in the gastroesophageal junction [9]. In the present study, one of the positive cases for HER2 was located in the gastroesophageal junction, and the other in the stomach body. Both samples presented a heterogeneous staining pattern, which commonly occurs for HER2 in GC [10–11, 19]. The IHC was subsequently confirmed by FISH analysis in the 50 GC cases, with 100% concordance between both tests. In fact, the latter experimental approach revealed that the two cases in which overexpression of HER2 was observed at the protein level, presented amplification of the gene encoding for this oncoprotein. Presumably, amplification of the gene *ERBB2* is a biological indication of a more aggressive cancer phenotype; it has been shown that HER2 overexpression, due to amplification of this gene, in GC is associated with worse prognosis [21–23].

Tests for the diagnosis (IHC) and confirmation (FISH) of HER2 overexpression can lead to variability in the obtained results due to factors such as the conditions in which the tests are performed, and interpretation criteria. Therefore, thorough standardisation and validation are of paramount importance when implementing these tests in a laboratory [22]. Thus, obtaining robust and reliable results depend on a series of factors, some inherent to the correct execution of the IHC or FISH protocols, others related to the collection and processing of tissue samples. For this study, several controls were implemented to warrant the reproducibility of the IHC or FISH tests. However, there were factors we could not control for, namely, the time elapsed between taking the tissue sample and its fixation in formalin and the quality of the latter [10]. A factor that we did pay attention to in this study was the tissue deparaffinisation of the tissue slides prior to IHC and FISH procedures. This is an important process since insufficient deparaffinisation can lead to inappropriate labelling/hybridisation and, ultimately, incorrect results [10].

Among the limitations of this study was the use of breast cancer tissue of known HER2 status as controls, mainly because of the differences in HER2 IHC staining patterns between breast and GC. Notwithstanding this, we utilised breast cancer tissues merely as experimental control; that is, to ensure that the IHC staining procedure was properly done. Also, we did not have GC tissue of known HER2 status at that time to use as the experimental control. The control breast cancer tissue was, in addition, used in the FISH tests, as a confirmatory method of the adequate performance of the test, in addition to the control slides provided by the manufacturers as part of the FISH kit. Another limitation in our study pertains to the data obtained from the clinical records; the low number of patients for whom HER2 analyses were requested and the nonperformance of FISH as a confirmatory test for all cases with IHC 2+. Finally, we did not repeat the IHC and FISH analyses in all 50 GC tissue specimens, but only the two samples in which IHC against HER2 revealed positive staining/amplification; this could also be considered as a limitation of our study.

## Conclusion

The present study suggests that the prevalence of GC cases with high expression of the HER2 oncogene in Costa Rica could range between 4% and 8%. Although we provide, for the first time, an estimation of the prevalence of HER2-overexpressing cases of advanced GC in Costa Rica, by no means should this be interpreted as the real prevalence. As already alluded to, most of the GC cases were not subjected to evaluation of the HER2 status, as evidenced in the clinical records; also, the clinical records retrieved for this study only represent a specific period of time, and the 50 surgical specimens used to perform IHC and FISH were not randomly chosen. Notwithstanding this, the prevalence here reported is consistent with other reports from various latitudes. Given the very high mortality rate of GC in Costa Rica, we suggest that HER2 testing should be considered at least in those cases that, according to clinical-pathological characteristics, are more likely to be positive for this oncoprotein. We consider the latter as a potential strategy to adopt in countries in which the health budget is limited, as this information is crucial to provide clinical benefit to GC patients with the highest risk of overexpressing HER2.

## Conflicts of interest

The authors declare that they have no conflicts of interest.

## Funding

Vicerectoria de Investigación, Universidad de Costa Rica, MAKOL.

## Figures and Tables

**Table 1.** Expression of HER2 in patients with advanced GC according to the information reported in the clinical files.

Diagnostic test	n
HER 2 Inmunohistochemistry	
0–1+	53
2+	5
3+	4
HER 2 FISH	
Analysed	
HER2/CEN17 > 2	1
HER2/CEN17 < 2	0
Not analysed	61

**Table 2.** Clinical-pathological characteristics of GC patients from whom the tissue samples were used to carry out IHC and FISH.

Variable	Number of patients (%)
Average age (age ± SD)	63.5 ± 13.2 [range: 29–85]
Sex Male Female	34 (68) 16 (32)
Histological type Well-differentiated Moderately differentiated Poorly differentiated Mucinous Hepatoid Mixed Signed ring Medullary	12 (24) 14 (28) 19 (38) 1 (2) 1 (2) 1 (2) 1 (2) 1 (2)
Location Cardias Body Antrum Pylorus Body-antrum Gastroesophageal junction	15 (30) 12 (24) 16 (32) 1 (2) 1 (2) 5 (10)
